# Association mining of mutated cancer genes in different clinical stages across 11 cancer types

**DOI:** 10.18632/oncotarget.11392

**Published:** 2016-08-19

**Authors:** Wangxiong Hu, Xiaofen Li, Tingzhang Wang, Shu Zheng

**Affiliations:** ^1^ Cancer Institute (Key Laboratory of Cancer Prevention and Intervention, China National Ministry of Education), The Second Affiliated Hospital, Zhejiang University School of Medicine, Hangzhou, Zhejiang 310009, China; ^2^ Zhejiang Institute of Microbiology, Hangzhou, Zhejiang 310012, China

**Keywords:** Apriori algorithm, association analysis, frequent mutation gene sets, mutation, Pan-Cancer

## Abstract

Many studies have demonstrated that some genes (e.g. *APC*, *BRAF*, *KRAS*, *PTEN*, *TP53*) are frequently mutated in cancer, however, underlying mechanism that contributes to their high mutation frequency remains unclear. Here we used Apriori algorithm to find the frequent mutational gene sets (FMGSs) from 4,904 tumors across 11 cancer types as part of the TCGA Pan-Cancer effort and then mined the hidden association rules (ARs) within these FMGSs. Intriguingly, we found that well-known cancer driver genes such as *BRAF*, *KRAS*, *PTEN*, and *TP53* were often co-occurred with other driver genes and FMGSs size peaked at an *itemset* size of 3∼4 genes. Besides, the number and constitution of FMGS and ARs differed greatly among different cancers and stages. In addition, FMGS and ARs were rare in endocrine-related cancers such as breast carcinoma, ovarian cystadenocarcinoma, and thyroid carcinoma, but abundant in cancers contact directly with external environments such as skin melanoma and stomach adenocarcinoma. Furthermore, we observed more rules in stage IV than in other stages, indicating that distant metastasis needed more sophisticated gene regulatory network.

## INTRODUCTION

Cancer is driven largely by somatic ‘driver mutations’ that accumulate in the genome [1, 2]. And different cancers often result from different combinations of driver genes [3, 4]. So far, hundreds of cancer driver genes have been annotated in COSMIC [5], although insightful, underlying interaction of these driver genes in specific cancer genome remains unclear. Previous study has showed exclusivity and co-occurrence between significantly mutated genes (SMGs) in different tumor types [6]. Nevertheless, co-occurrence of more than two SMGs, which can help us to better understand tumorigenesis and tumor evolution, is not explored in their study.

Frequent items sets (for short *itemsets*) are lists of items that commonly appear together. Association rules (ARs) suggest that a strong relationship exists between two items. Mining ARs is first introduced by Agrawal *et al*. and is familiar with market basket analysis [7]. In cancer genome, we also speculate that some frequent mutation genes (e.g. *APC*, *TP53*, *PTEN*) may result from mutation of other gene(s) and *vice versa*. Further, as different cancer pathologic stages showed distinct clinical characteristics [8], we assume that different pathologic stages vary greatly in the presence and absence of specific frequent mutation gene sets (FMGSs) and ARs owing to heterogeneous mutation profiles. The current widely used driver gene identification method is based on whole cancer genome mutation recurrence frequency, which may underestimate the driver genes in each stage because of variation in stage background [6, 9]. Thus, it is necessary to explore the FMGSs and accompanying ARs in a stage-dependent manner.

In this study, we focused on the identification of FMGSs and their contribution to co-occur of each other by using Apriori algorithm in American Joint Committee on Cancer (AJCC) four stages across 11 cancers (Breast invasive carcinoma (BRCA), Colorectal cancer (CRC, Colon adenocarcinoma (COAD)/Rectum adenocarcinoma (READ)), Head and neck squamous cell carcinoma (HNSC), Kidney renal clear cell carcinoma (KIRC), Liver hepatocellular carcinoma (LIHC), Lung adenocarcinoma (LUAD), Ovarian serous cystadenocarcinoma (OV), Skin cutaneous melanoma (SKCM), Stomach adenocarcinoma (STAD), Thyroid carcinoma (THCA), Uterine corpus endometrial carcinoma (UCEC)) as part of the Cancer Genome Atlas (TCGA) Pan-Cancer effort. Interestingly, we found that well-known cancer driver genes such as *BRAF*, *KRAS*, *PTEN*, and *TP53* were often co-occurred with other driver genes and FMGSs size peaked at an *itemset* size of 3∼4 genes. Furthermore, AR learning in four stages showed that both AR number and pattern differed greatly, especially in stage IV. It is thus tempting to believe that tumor distant metastasis needs more sophisticated gene regulatory network. Deciphering gene relationships (possibly provide a direction of action) may assist biomedical research in determining the underlying cause of cancer and developing specific gene-targeting treatments.

## RESULTS

### Overview of the mutation profiles in 11 cancers

Mutational profiles of the 11 cancers (BRCA-137,734, CRC-170,587, HNSC-355,587, KIRC-67,638, LIHC-1,590,829, LUAD-562,793, OV-27,651, SKCM-803,270, STAD-530,769, THCA-31,863, UCEC-240,547; numbers indicated the total point mutations and small insertions/deletions) accumulated from whole exome sequencing method were collected from TCGA project. Silent mutations and mutations refer to RNA were removed. The retained mutation profiles (BRCA-103,596, CRC-124,363, HNSC-251,760, KIRC-52,225, LIHC-1,428,996, LUAD-419,132, OV-20,823, SKCM-521,755, STAD-395,078, THCA-20,265, UCEC-182,586) were used for refining the mutated genes in a total of 5,083 tumors. Now the mutated genes in single tumor were counted just like the transaction in market analysis; that is, each transaction in tumor had a unique patient ID (BRCA-1,000, CRC-388, HNSC-523, KIRC-548, LIHC-199, LUAD-515, OV-463, SKCM-368, STAD-385, THCA-446, UCEC-248) and contained different subset of the genes. To obtain high-confidence mutated transaction, transaction (i.e. patient) with less than 10 mutation genes (hypomutation) or more than 5,000 mutation genes (hypermutation) were excluded. This led to the retention of 4,904 tumors across 11 cancer types: BRCA-978 (median mutated genes = 39), CRC-383 (median = 85), HNSC-523 (median = 127), KIRC-478 (median = 58), LIHC-193 (median = 165), LUAD-514 (median = 214.5), OV-431 (median = 43), SKCM-367 (median = 303), STAD-380 (median = 127), THCA-417 (median = 22), UCEC-240 (median = 90), respectively. In order to compare the FMGSs and ARs in different stages, clinical information of each patient was added in the dataset via the unique patient ID. The compiled data were subject to FMGS interrogation and rules mining.

### FMGSs in four clinical stages across 11 cancers

Previous studies put much emphasis on the mutational landscape in diverse cancers [6, 10]. However, the FMGSs (i.e. the co-occurrence of specific genes) and their variation in different stages are rarely explored. Systematically mutation-centric analysis is hampered by a lack of enough dataset prior to the TCGA project [11]. Here we conducted an in-depth FMGSs identification and AR mining among four clinical stages across 11 tumor types to investigate hidden relationships of mutational genes. Considering reliability of ARs and gene mutation frequency in cancer [3, 12–14], the default support for *k*-1∼n (n ≥ 2) FMGS was set at 0.1 (gene mutation account for more than 10% of cancer patients) and confidence of a rule was set to 0.9. The high confidence value can help to eliminate the pseudo-strong rules since a single gene mutation rate rarely exceeds 90%.

**Figure 1:**
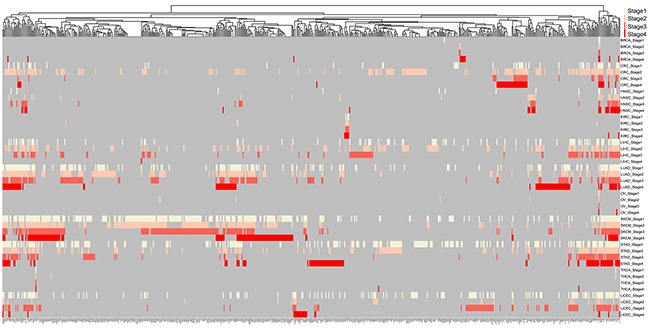
Clustering of 620 mutated genes in at least two cancer stages Milk white, pink, orange, red, and grey indicate specific genes mutated in stage I, stage II, stage III, stage IV, and NA, respectively.

**Figure 2:**
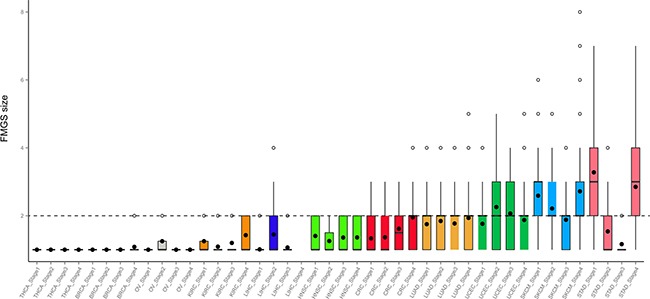
Distribution of FMGS size in four stages across 11 cancer types Dashed grey line denotes FMGS size of two genes across cancer types.

**Figure 3:**
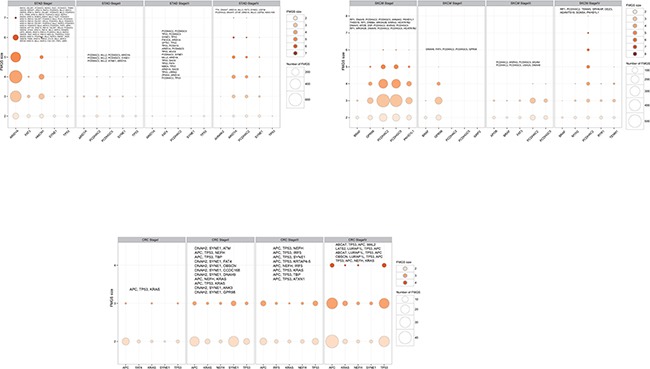
Number of FMGS with different size among the top five highest mutation genes in each clinical stages in selected cancers: CRC, SKCM, and STAD The bubble size corresponding to the number of FMGS and the color corresponding to the FMGS size, respectively. The largest FMGS in each stage was shown in the top of bubble plot and genes that identical to the top five highest mutation ones were shaded in bold font.

Totally, 1,156 unique *k*-1 FMGSs were identified in all 44 cancer stages (11×4) and 620 *k*-1 FMGSs were present in at least two different stages. Clustering the 620 *k*-1 FMGSs showed that there existed great heterogeneity among different cancer stages (Figure 1). Briefly, more *k*-1 FMGSs were found in stage I than in other three stages in SKCM and STAD. In CRC and LIHC, three-fold more *k*-1 FMGSs were observed in stage II than in other three stages (Figure 1). Though FMGSs were rarely observed in four cancers (BRCA, KIRC, OV, and THCA; Table 1, Figure 1, Figure 2), their key driver genes and high mutation frequencies were in accordance with previous Pan-cancer studies (e.g. *TP53* (30.9%, 35.5%, 32.1%, 53.3% corresponding to stage I, II, III, and IV, respectively) and *PIK3CA* (37.7%, 34%, 32.6%, 33.3%) in BRCA, *VHL* (43.7%, 30.8%, 42.4%, 41.4%) and *PBRM1* (37.8%, 30.8%, 36.4%, 35.7%) in KIRC, *TP53* (100%, 85.7%, 84.3%, 90%) in OV, and *BRAF* (57.3%, 40.8%, 71.6%, 75%) in THCA; Supplementary Table S1). As for other cancer types, the well-known driver genes (e.g. *TP53*, *PTEN*, *PIK3CA*) were frequently co-occurred with other cancer genes; that is, constitute as larger FMGSs. For example, in STAD, *ARID1A* frequently co-occurred with *PCDHAC2*, *PCDHGC5*, *MLL2*, *HERC2*, *etc* (Figure 3A). *RP1*, *PCDHAC2* had pretty high mutation rate in SKCM, and often co-occurred with *PCDHGC5*, *DNAH9*, *MROH2B*, *etc* (Figure 3B). In CRC, mutation of *APC*, *TP53*, *KRAS* were frequently co-occurred, and *TBP*, *NEFH*, *SYNE1* were often mutated together with *APC* and *TP53*, respectively (Figure 3C, Supplementary Table S2).

**Table 1:** The number^a^ of FMGS and AR mined in 44 cancer stages

	Stage I	Stage II	Stage III	Stage IV
BRCA	3/3/0	4/4/0	4/4/0	12/10/4
CRC	84/57/4	320/250/2	68/36/0	193/68/7
HNSC	37/22/1	43/33/0	53/35/0	47/30/2
KIRC	4/2/0	11/9/0	5/3/0	7/3/0
LIHC	102/100/0	342/230/176	123/110/0	0/0/0
LUAD	479/326/13	651/401/19	382/226/10	357/147/99
OV	1/1/0	4/2/0	1/1/0	1/1/0
SKCM	3,923/1,628/1,017	1,606/986/62	641/403/18	863/173/781
STAD	4,318/647/3,081	286/198/8	104/89/0	576/111/682
THCA	2/2/0	2/2/0	1/1/0	1/1/0
UCEC	397/209/0	113/27/73	477/163/76	87/31/52

a Numbers before and after the slash indicate the number of total FMGS, unique FMGS, and AR, respectively.

As described above, in BRCA, KIRC, OV, and THCA, FMGSs were rarely observed and the FMGS size was commonly < 3 (Figure 2). By contrast, in other cancer types such as SKCM and STAD, the FMGSs size peaked at an *itemset* size of 3. Besides, as many as 8 genes that participated in several core cellular pathways were involved in SKCM tumorigenesis and development. This result indicates that the initiation and development of SKCM requires more gene aberration. (Figure 3). The largest FMGS identified in all these cancer stages contained 8 genes (*k* = 8; *RP1*, *PCDHAC2*, *TENM3*, *SPHKAP*, *ODZ3*, *ADAMTS18*, *SCN5A*, *PKHD1L1*) found in SKCM-stage IV. Further, the FMGSs size also differed greatly in four clinical stages of the same cancer. For example, many more FMGSs and larger FMGS size were observed in stage I/IV compared to stage II/III in STAD (4,318/576 vs. 286/104 items) and SKCM (3,923/863 vs. 1,606/641 items), respectively (Figure 2, Table 1). Collectively, our data revealed that different cancer types and even different stages of the same cancer had distinct driver gene patterns, which should be born in mind in future targeted cancer therapy.

### Extraction of ARs in different clinical stages

**Figure 4:**
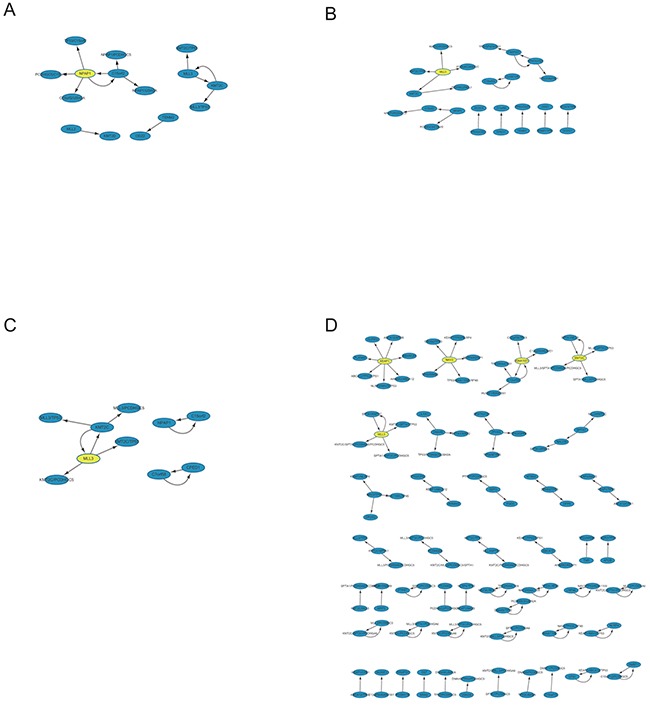
Network-based visualization of the ARs in LUAD four stages Evidently many more rules were observed in stage IV relative to the other three stages. We reasoned that more sophisticated gene association pattern is needed for tumor distant metastasis.

To further elucidate the putative correlation of these high mutation genes within FMGSs, Apriori algorithm that based on conditional probabilistic theory was used for mining ARs in abovementioned cancer stages. Once the frequent *k*-*itemsets* (i.e. k-FMGS) were found, we converted them into rules by splitting the *k*-*itemsets* (k ≥ 2) into *antecedent* (also known as LHS) and *consequent* (also known as RHS). A rule was defined as an implication of the form *X* (*antecedent*) ⇒ *Y* (*consequent*), meaning *X* mutation probably lead to the occurrence of *Y*. Since ARs were based on FMGS, ARs were rarely observed in four cancers (BRCA, KIRC, OV, and THCA; Table 1). We then focused attention on the other 7 cancers (CRC, HNSC, LIHC, LUAD, SKCM, STAD, UCEC). Intriguingly, in accordance with FMGS, we found that ARs also differed greatly among the four stages. For instance, as many as 1,017 rules were generated in SKCM stage I vs. 18 rules in stage III. And no valid rule was found in CRC stage III, STAD stage III, UCEC stage I, HNSC stage II and III, LIHC stage I, III, and IV. In LUAD, only 13, 19, and 10 interesting rules were generated in stage I∼III (Figure 4A∼C), in sharp contrast with 99 rules generated in stage IV (Figure 4D, Supplementary Table S2).

Interestingly, we found that a subset of LHSs and corresponding RHSs could swap interchangeably; that is, LHS in a rule could become RHS in another rule and *vice versa* (e.g. a pair of rules *MLL3* ⇒ *KMT2C* and *KMT2C* ⇒ *MLL3* in LUAD stage I; Supplementary Table S2). These interchangeable rules (*X* ⇔ *Y*) were extremely useful in cancer treatment because inhibition or restoration of either LHS or RHS will still induce their mutation and possibly accompany cancer development. As such, we should inhibit or restore LHS and RHS simultaneously by using combination of drugs or other measures.

We should bear in mind that an AR (*X* ⇒ *Y*) did not always uncover a causal relationship between *X* and *Y*. There may be other hidden variables that cannot be deduced from the rule. For example, rule *RNF43* ⇒ (*OBSCN*, *C14orf43*, *PTEN*, *NEB*) represent mutation of *RNF43* may be associated with the mutation of (*OBSCN*, *C14orf43*, *PTEN*, *NEB*). Instead of pointing dependencies among these four genes, a hidden node, UCEC stage II, was the hidden variable concerned with the mutation of these four genes.

## DISCUSSION

As an unsupervised learning method, association analysis with the Apriori algorithm can be a powerful method to explore the underlying relationship between two items under large dataset [7]. To the best of our knowledge, this is the first time that this method is used in somatic mutation data mining. Although a lower support can generate more FMGSs and rules, a cutoff of 0.1 was chosen to avoid inherent mutational noise and statistical error.

Since tumor heterogeneity prevails in cancers [15, 16], the mutational landscape may differ considerably among different clinical stages of the same tumor. Indeed, the number and constitution of FMGS differed greatly among different stages (Table 1, Supplementary Table S1). Recently, a study reports that *RNF43* is frequently mutated in colorectal and endometrial cancers [17]. In our study, we further determined that the high mutation rate of *RNF43* was confined to stage I/II in CRC and UCEC (Supplementary Table S1). Moreover, they also speculate that stomach cancer also harbors frequent mutations in *RNF43* [17]. Indeed, we observed that high mutation rate existed in all four clinical stages in STAD (Supplementary Table S1). On the contrary, in CRC, high mutation of *LATS2* was observed in stage III/IV, but not in stage I/II. From this point of view, the so-called driver genes may be underestimated via the current widely used recurrence frequency method due to the distinct mutational background in different clinical stages. And some genuine cancer driver genes were probably neglected by previous driver gene identification method based on the whole cancer genomes. In parallel, underlying stage-based ARs may also be masked by using the whole cancer genomes.

As mentioned earlier, few FMGSs and accompanying ARs were observed in BRCA, KIRC, OV, and THCA. We have attempted to lower the support to 0.05, the results, however, remained basically unaltered. For BRCA, although we stratified the samples into currently widely used five main molecular subtypes, namely basal-like, Her2 positive, luminal A, luminal B, and normal breast-like [13, 18, 19], similar results still held (data not shown). One explanation is that the mutation profile is quite heterogeneous in these cancers. Another one can be ascribed to their intrinsic low mutation frequencies compared with other cancers [6, 20]. In addition, BRCA, OV, and THCA are all endocrine-related cancers, we reason that hormone imbalance, not gene mutation, is the major cause of cancer occurrence and progression.

As for the other cancer types, FMGS size peaked at an *itemset* size of 3∼4, suggesting that only small number of driver genes were sufficient to induce tumorigenesis and cancer progression, which was in accordance with previous studies [21, 22]. Quite unexpectedly, though some genes (e.g. *BRAF* in SKCM, *TP53* in STAD and UCEC) had high mutation rates, their FMGSs size was < 3 (Figure 2). And their corresponding ARs were also very sparse (Supplementary Table S2), suggesting that they may act as key driver genes to initiate cancer without interacting with other genes.

With the new era of big data coming, the need to extract and link underlying knowledge from large databases is increasing. Extracting interesting ARs from gene mutation datasets is very important in identifying the cause of diseases including cancer [23]. To date, only single, or at most two mutual genes of mutation profile have been drawn. In this study, a compendium of FMGSs and accompanying association patterns in four clinical stages were explored across 11 cancers. Though the somatic mutation mode is quite different from each other, it may shed light on the occurrence, progression of cancer, and contribute to cancer treatment. In conclusion, FMGSs and ARs identified in this study are useful for cancer treatment such as combination drug therapy, which now is imperative to precision medicine that has received great attention.

## MATERIALS AND METHODS

### Data retrieval and processing

All cancer somatic mutation data and clinical information were downloaded from the TCGA data portal (02/03/2015). Silent mutation and RNA mutation were discarded. Then the remaining mutation sites were subjected to evaluate whether an amino acid substitution affects protein function by SIFT [24] and PolyPhen-2 [25], and only predicted harmful sites were retained in the file. Retained mutation profiles in each cancer were used for refining the mutated genes in a total of 5083 tumors. Then samples with fewer than 10 mutation genes (hypomutation) or more than 5000 mutation genes (hypermutation) were also discarded. Lastly, clinical information of each patient was added right after mutational genes via the unique patient ID.

### Finding FMGS in four clinical stages

The number of patients in these cancer types (commonly > 300 samples) was large enough to stratify patients into four stages (stage I, stage II, stage III, and stage IV). Then Apriori algorithm was used for exploring the FMGSs and ARs of mutation genes in each stage. Let *G* = {*g_1_, g_2_, …, g_n_*} be a set of *n* genes (e.g. ensemble gene sets of human) called *items*. Let *D* = {*t_1_, t_2_*, …, *t_m_*} be a set of transactions (patients in TCGA) called the database. Each transaction in *D* has a unique patient ID and contains a subset of the genes in *G*. To find frequent sets of items (for short *itemsets*) quickly (without enumeration of all subsets of *items*), the Apriori algorithm uses the hypothesis that if {*g_1_*, *g_2_*, *g_3_*} is frequent (satisfy support threshold), all its subsets {*g_1_, g_2_*}, {*g_1_, g_3_*}, and {*g_2_, g_3_*} should be frequent as well. In other words, if a two-*itemset* {*g_1_, g_2_*} is known not to be frequent, all its supersets (including *g_1_* or *g_2_*) need not to be checked and can be pruned.

Starting by finding the frequent one-*itemsets* (*k* = 1), we generate candidate *k*+1 *itemsets* iteratively and check if they satisfy the support threshold. Note that the number of candidate *itemsets* will decrease rapidly as *k* increases. A total of *n*+1 iterations are needed if the largest *itemset* has *n items*.

### ARs extraction in four clinical stages

Once we find the frequent *k-itemsets*, we convert them into rules by splitting the *k-itemsets* (k ≥ 2) into *antecedent* (Gene_x_, hereafter *X*) and *consequent* (Gene_y_, hereafter *Y*). A rule is defined as an implication of the form *X* ⇒ *Y* where *X*, *Y* ⊆ *I* and *X* ∩ *Y* = ∅, meaning mutation of *X*probably lead to *Y*mutation. The *itemsets X* and *Y* are called *antecedent* (left-hand-side or LHS, one gene or more) and *consequent* (right-hand-side or RHS, one gene or more) of the rule. We start by putting a single gene in the *consequent* and *k*−1 genes in the *antecedent*. An interesting AR is a rule that surpasses a user-specified minimum support and minimum confidence threshold. Support (*X*) is defined as the proportion of patients in each tumor stage that contains the *itemset* and the confidence of a rule is defined as follows

$$\text{Confidence}\left(X\Rightarrow Y\right)=P\left(Y|X\right)=\frac{P\left(X,Y\right)}{P\left(X\right)}=\frac{\text{supp}\left(X\cup Y\right)}{\text{supp}\left(X\right)}$$

Therefore, an AR *X* ⇒ *Y* will satisfy:

Supp(X∪Y)≥σ

and

Conf(X⇒Y)≥δ

where σ and δ are user-defined manually.

By default, to obtain reliable rules, minimum support (σ) was set at 0.1 and confidence (δ) was set at 0.9 unless otherwise specified. For stages with patients less than 30, support was elevated to 0.15 (group-based minimum support). In SKCM, higher support was observed overall and the default support was set at 0.15 and 0.2 for stage IV because it only involved 19 patients. A lower support or confidence can give rise to more FMGSs and rules, but will also lead to spuriously significant findings. In the meantime, the confidence of a rule *X* ⇒ *Y* does not measure the real strength of the correlation and implication between *X* and *Y* and it sometimes can be deceiving. One simple way to weigh the correlation of *X* and *Y* is *lift*.

$$\text{Lift}\left(X\Rightarrow Y\right)=\frac{P\left(X,Y\right)}{P\left(X\right)P\left(Y\right)}=\frac{P\left(Y|X\right)}{P\left(Y\right)}=\frac{\text{supp}\left(X\cup Y\right)}{\text{supp}\left(X\right)\text{supp}\left(Y\right)}$$

In brief, the occurrence of *Y* is independent of the occurrence of *X* if *P*(*X* ∪ *Y*) = *P*(*X*)*P*(*Y*); otherwise, *Y* and *X* are bond and correlated as events. And the *lift* value < 1 and > 1 indicate the occurrence of *X* is negatively or positively correlated with the occurrence of *Y*, meaning that the occurrence of *X* likely leads to the absence or occurrence of *Y*, respectively.

Additionally, since prevalent mutational heterogeneity in cancer and *lift* can be easily influenced by the number of null-transactions ($\bar{XY}$). Here, in combination with *lift* filtering (*lift* > 2), we used Kulczynski measure (Kulc) for pattern exploration. Rules with Kulc > 0.7 were retained in the final visualization.

$$\text{Kulc}\left(X\Rightarrow Y\right)=\frac{1}{2}\left(P\left(X|Y\right)+P\left(Y|X\right)\right)=\frac{1}{2}\left(\text{conf}\left(X\Rightarrow Y\right)+\text{conf}\left(Y\Rightarrow X\right)\right)$$

### Data visualization

Unless otherwise stated, data visualization was performed in R (version 3.0.2) and ggplot2 package [26]. ARs were visualized in network format by Cytoscape (v3.2.1) [27].

## SUPPLEMENTARY MATERIALS TABLES






