# Supplementary material for: Association mining of mutated cancer genes in different clinical stages across 11 cancer types
Source: Oncotarget. 2016 Aug 19;7(42):68270–7. doi: 10.18632/oncotarget.11392 (PMC5356553; doi:10.18632/oncotarget.11392)
Supplement: Supplementary file 1 [file oncotarget-07-68270-s001.pdf]

Association mining of mutated cancer genes in different clinical stages across 11 cancer types

SUPPLEMENTARY TABLES

Supplementary Table S1: FMGS identified in 11 cancer types.

See Supplementary File 1

Supplementary Table S2: Association rules generated in CRC, HNSC, LIHC, LUAD, SKCM, STAD, UCEC.

See Supplementary File 2
